# The number and size of Lugol‐voiding areas were reduced by pneumatic dilation in a patient with achalasia and esophageal cancer

**DOI:** 10.1002/jgh3.12244

**Published:** 2019-08-18

**Authors:** Shinwa Tanaka, Hirofumi Abe, Ryusuke Ariyoshi, Hiroya Sakaguchi, Taro Oshikiri, Tetsu Nakamura, Yoshiko Nakano, Yoshinori Morita, Takashi Toyonaga, Eiji Umegaki, Hiroshi Yokozaki, Yoshihiro Kakeji, Yuzo Kodama

**Affiliations:** ^1^ Divisions of Gastroenterology, Department of Internal Medicine Kobe University Graduate School of Medicine Kobe Japan; ^2^ Divisions of Gastrointestinal Surgery, Department of Surgery Kobe University Graduate School of Medicine Kobe Japan; ^3^ Divisions of Pathology, Department of Pathology Kobe University Graduate School of Medicine Kobe Japan

**Keywords:** achalasia, esophageal cancer, Lugol, Lugol‐voiding area, pneumatic dilation

## Abstract

Achalasia is a rare benign esophageal motility disease caused by the impaired relaxation of the lower esophageal sphincter, which results from nerve damage. Patients with achalasia are known to have a high risk of esophageal cancer. Here, we present the case of a patient with achalasia and esophageal cancer in whom the Lugol‐voiding areas (LVAs) could be improved by pneumatic dilation and the extending area of esophagus cancer could become clear. In achalasia patients, LVAs are modified by inflammation and appear wider than their actual size. Moreover, some parts of LVAs in achalasia patients might be reversible by treatments that improve delayed emptying. When the spread of esophagus cancer is unclear due to the detection of numerous LVAs by Lugol chromoendoscopy, the treatments that improve delayed emptying first may be effective in accurately diagnosing the extending area of esophagus cancer.

## Graphical abstract

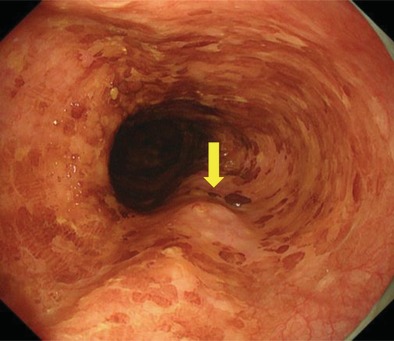

In achalasia patients, Lugol‐voiding areas (LVAs) are modified by inflammation and appear wider than their actual size. Moreover, some parts of LVAs in achalasia patients might be reversible by treatments that improve delayed emptying.

## Introduction

Achalasia is a primary esophageal motility disorder characterized by the absence of peristalsis and defective relaxation of the lower esophageal sphincter (LES), resulting in impaired bolus transport and the retention of food in the esophagus. Its incidence has been reported to be approximately 1/100 000 worldwide.^1^ Although achalasia is a benign disease and has a good prognosis, the co‐occurrence of esophageal cancer and achalasia is a serious concern, and the prognosis of patients who develop such a disease remains poor. Previous studies have reported that the incidence of concomitant esophageal cancer and achalasia ranges from 0.5 to 8.6%.^2^ In a recent study, Meijssen *et al*. reported that the risk of squamous cell carcinoma (SCC) was >33‐fold higher in achalasia patients than in the general population.^3^ Persistent esophageal distension combined with the retention of food and fluids, bacterial overgrowth, and impaired clearance of regurgitated acid and gastric contents, which can lead to chronic inflammation, are considered to be causes of carcinoma.^4^ Performing chromoendoscopy in combination with Lugol staining remains the gold‐standard technique for detecting superficial esophageal SCC.^5^ Clinically, Lugol chromoendoscopy (LCE) can be used to visualize epithelial changes, such as Lugol‐voiding areas (LVAs), as dysplastic epithelia are not stained with Lugol iodine solution and appear white or pink, whereas normal epithelia are stained brown. Conversely, LVA can also occur due to inflammation, and in cases of concurrent cancer and inflammation, the presence of LVA often makes it difficult to determine the extent to which the esophageal cancer has spread. We present the case of a patient with achalasia and esophageal cancer, in whom the number and size of LVA were reduced by pneumatic dilation, which clarified the extent of the esophageal cancer.

## Case report

**Figure 1:**
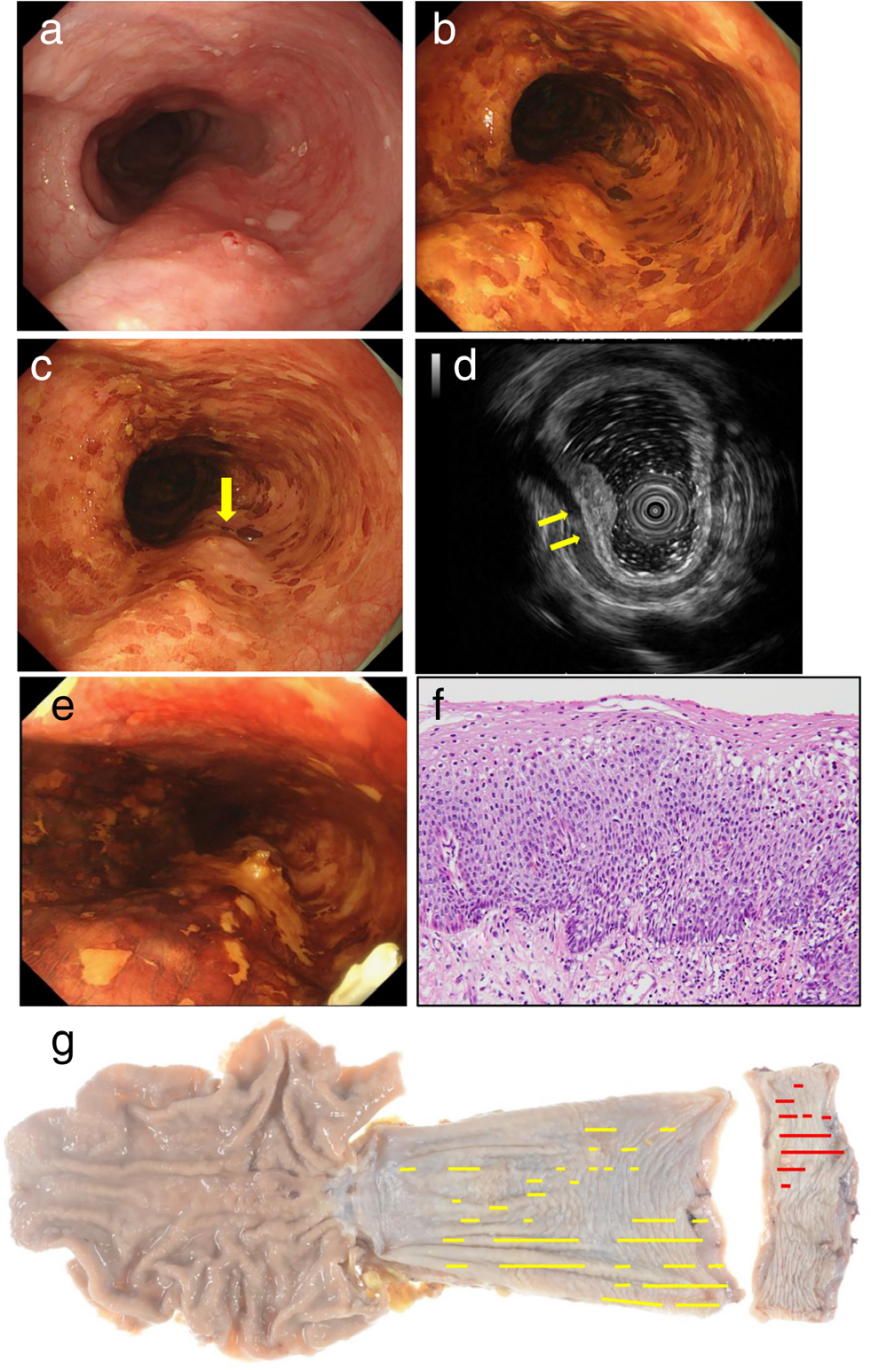
Esophageal cancer associated with achalasia. (a) An elevated lesion was noted in the upper thoracic esophagus. (b) During Lugol chromoendoscopy, Lugol‐voiding areas (LVA) were seen around the entire circumference of the lumen surrounding the elevated lesion. (c) The color of the elevated lesion changed to pink (yellow arrow). (d) After the pneumatic dilation procedure, the LVA surrounding the elevated lesion were limited to half of the circumference of the esophagus. (e) Endoscopic ultrasonography showed that the submucosal layer was thin and irregular in the elevated region (yellow arrow). (f) Diffuse inflammatory cells, such as neutrophils and eosinophils, were noted in the esophageal mucosa, but no cancer was detected. (g) The macroscopic specimen is shown (red: Squamous cell carcinoma, yellow: High‐grade intraepithelial neoplasia).

A 72‐year‐old male with an approximately 3‐year history of progressive dysphagia and regurgitation and a baseline Eckardt score of 6 was referred to our hospital. He had consumed 50 g of alcohol per day for 50 years and smoked 20 cigarettes per day for 50 years. Esophagogastroduodenoscopy (EGD) showed fluid retention, a dilated esophagus, and a tight esophagogastric junction (EGJ). Moreover, multiple reddened areas were noted throughout the esophagus, and an elevated lesion (diameter: 10 mm) was detected in the upper thoracic esophagus (Fig. 1a). Narrow‐band imaging (NBI) demonstrated multiple diffuse brownish areas, which made it difficult to determine the extent to which the elevated lesion was cancerous. After the application of Lugol dye solution, numerous well‐defined, irregularly shaped LVAs appeared throughout the entire esophageal mucosa. The LVA covered the entire circumference of the lumen surrounding the elevated lesion, and the color of the elevated lesion changed to pink (Fig. 1b,c). Based on a pathological examination of a biopsy specimen obtained from the elevated lesion, a diagnosis of SCC was made. Endoscopic ultrasonography (EUS) demonstrated that the lesion had invaded deep into the submucosa; that is, the submucosal layer was thin and irregular in the elevated region (Fig. 1d). An esophagogram showed delayed esophageal emptying and narrowing at the LES. High‐resolution manometry (HRM) demonstrated panesophageal pressurization and impaired LES relaxation. We diagnosed the patient with nonsigmoid achalasia type II according to the Chicago classification. With respect to the depth of the esophageal cancer, we made a diagnosis of deep submucosal invasion based on the findings of EUS and the fact that the lesion exhibited a convergent fold pattern. No lymph node metastasis or distant metastasis was detected on computed tomography or ^18^F‐2‐fluoro‐2‐deoxy‐d‐glucose‐positron emission tomography. The patient's preoperative clinical findings were classified as cT1b N0 M0 Stage I according to the 10th edition of the Japanese Classification of Esophageal Cancer (JCEC) and as cT1, N0, M0, cStage IA according to the 7th tumor‐node‐metastasis (TMN) classification developed by the International Union Against Cancer (UICC). We planned to treat this case surgically. However, the patient's dysphagia suddenly worsened a week after the EGD was performed, and he was unable to drink any liquid. He was immediately hospitalized and underwent pneumatic dilation. A single pneumatic dilation session was performed with a 30 mm Rigiflex II single‐use achalasia balloon dilator (Boston Scientific, Marlborough, MA, USA). The EGJ was dilated by 30 mm for 1 min at 18 psi. After the dilation procedure, the patient's dysphagia improved, and he was able to eat anything he wanted. EGD was performed again 36 days after the pneumatic dilation procedure. The elevated lesion had increased in size, and LCE revealed that the LVA, which were present throughout the esophagus, had markedly decreased in size, and the LVAs surrounding the elevated lesion were limited to only half of the circumference of the esophagus (Fig. 1e). We did not administer any gastric secretion inhibitors before or after the pneumatic dilation procedure. The patient underwent thoracoscopic subtotal esophagectomy 42 days after the pneumatic dilation procedure. Reconstruction was performed via the retrosternal route using stomach tissue, and a cervical anastomosis was created through an incision in the neck. An examination of the resected specimen demonstrated that the tumor was located in the upper third of the esophagus. It measured 16 × 13 mm in size and was identified as type 0‐IIb + IIa. Pathologically, the lesion was classified as pT1b‐SM1 ly1 v1 pN0 pM0 pStage I according to the JCEC and T1b N0 M0 stage IA according to the UICC classification. Moreover, diffuse inflammatory cells, such as neutrophils and eosinophils, were noted in the esophageal mucosa, but no cancer was detected (Fig. 1f,g). The patient was discharged without any complications 25 days after the operation.

## Discussion

The Lugol staining technique is based on the fact that large amounts of glycogen, which is intensely stained by iodine, are present in the normal squamous epithelium. In contrast, dysplastic and carcinoma cells contain little or no glycogen, which results in the absence of staining.^6^, ^7^ Thus, LCE is still considered to be the best method for diagnosing esophageal SCC,^7^ whereas esophageal LVAs exhibit a wide variety of histologies, ranging from high‐grade neoplasia, such as SCC, to low‐grade neoplasia and even non‐neoplastic causes, such as inflammation.^6^

In patients with achalasia, persistent esophageal distension combined with the retention of food and fluids leads to chronic inflammation of the esophageal mucosa. In our case, diffuse inflammation was noted in the esophageal epithelium in the resected specimen. However, this inflammation was considered to be more severe than that seen before the pneumatic dilation procedure, which made the LVAs appear wider than they actually were. The number and size of the LVAs had decreased at 36 days after the pneumatic dilation procedure. These findings suggest that treatments which improve delayed emptying, such as pneumatic dilation, Heller myotomy, and per‐oral endoscopic myotomy, can eliminate changes caused by inflammation and that some inflammation‐derived LVAs are reversible. When a superficial esophageal cancer that is indicated for endoscopic submucosal dissection (ESD) is found in an achalasia patient, it can be challenging to determine whether the treatment of the achalasia or the esophageal cancer should be prioritized. Although a prolonged observation period might lead to the progression of the esophageal cancer, if the esophageal cancer could be observed for approximately 1 month, it would be better to administer treatment that improves delayed emptying before performing ESD so as to avoid excessive mucosal resection. In terms of the amelioration of LVAs, it is possible that the LVAs narrowed due to epithelial regeneration caused by Lugol staining‐induced irritation. However, in the current case, the second EGD procedure was performed 43 days after the initial EGD procedure. Changes in LVAs caused by Lugol staining‐induced irritation usually disappear within a month. Thus, we considered that Lugol staining‐induced irritation had little influence on our findings.

Patients that have suffered from achalasia for >20 years, have an enlarged esophagus, and exhibit esophageal flexure and marked food/fluid retention are considered to be at a particularly high risk of esophageal cancer.^8^ These patients should be monitored for SCC. Recently, a study reported that NBI is a simple and reliable technique for detecting SCC.^9^ However, SCCs were frequently misdiagnosed when NBI magnifying endoscopy was used to examine esophageal mucosae with multiple LVAs. On the other hand, the accuracy of LCE for diagnosing SCCs was reported to be markedly improved by also examining the esophagus for reddish or rose‐pink color changes, that is, the so‐called pink sign (PS).^10^ Performing LCE plus a PS assessment is a more reliable method for detecting esophageal cancer, even in cases involving a background esophageal mucosa that exhibits multiple LVAs.^10^ In our case, LCE demonstrated the PS in the elevated lesion. The region that exhibited the PS, which coincided with cancer that was diagnosed during the pathological examination, was hardly changed after the balloon dilation procedure. Thus, LCE should not be omitted in achalasia patients with multiple LVAs.

In conclusion, we encountered a case involving a patient with achalasia and esophageal cancer, in which pneumatic dilation ameliorated numerous LVAs and clarified the extent of the patient's esophageal cancer. In achalasia patients, LVAs are modified by inflammation, for example, they can appear wider than they actually are. Moreover, in such patients, it might be possible to normalize some LVAs using treatments that improve delayed emptying.
